# Supplementary material for: Decreased Physical and Daily Living Activities in Patients with Peripheral Arterial Disease on Hemodialysis
Source: J Clin Med. 2022 Dec 24;12(1):135. doi: 10.3390/jcm12010135 (PMC9821591; doi:10.3390/jcm12010135)
Supplement: Supplementary file 1 [file jcm-12-00135-s001.zip › jcm-2115107-supplementary.pdf]

Table S1. correlation of each indicator

	correlation coefficient																							
	①	②	③	④	⑤	⑥	⑦	⑧	⑨	⑩	⑪	⑫	⑬	⑭	⑮	⑯	⑰	⑱	⑲	⑳	㉑	㉒	㉓	㉔
① LSA	1																							
② ADL difficulty-basic ADL	.515**	1																						
③ ADL difficulty-ambulation	.498**	.743**	1																					
④ ADL difficulty-walking up or down	.471**	.777**	.827**	1																				
⑤ ADL difficulty-total	.530**	.875**	.949**	.941**	1																			
⑥ KCL total	-.533**	-.639**	-.692**	-.682**	-.728**	1																		
⑦ KCL physical	-.528**	-.673**	-.711**	-.710**	-.755**	.957**	1																	
⑧ KCL depression	-.368**	-.352**	-.421**	-.394**	-.426**	.765**	.544**	1																
⑨ ABI	.163*	0.083	.163*	.161*	.155*	-0.083	-0.103	-0.011	1															
⑩ GNRI	.346**	.280**	.176*	.216**	.228**	-.304**	-.300**	-.212**	.184*	1														
⑪ kt/v	-0.102	-0.005	-0.005	-0.042	-0.019	0.11	0.128	0.034	-0.131	-.177*	1													
⑫ Age	-.306**	-.458**	-.424**	-.390**	-.452**	.368**	.421**	0.129	-0.093	-.286**	0.067	1												
⑬ Water removal	.364**	.407**	.453**	.428**	.467**	-.475**	-.454**	-.365**	.178*	.334**	-0.125	-.403**	1											
⑭ BMI	.284**	.233**	.243**	.227**	.253**	-.322**	-.339**	-.177*	0.152	.420**	-.510**	-.378**	.524**	1										
⑮ Dialysis duration	-.339**	-.321**	-.191*	-0.143	-.220**	.173*	.172*	0.117	-0.056	-.246**	.235**	0.146	-0.143	-.292**	1									
⑯ Alb	.335**	.354**	.276**	.291**	.322**	-.266**	-.270**	-.169*	.174*	.773**	-0.022	-.410**	.248**	.239**	-.279**	1								
⑰ Hb	0.092	0.084	0.053	0.087	0.077	-0.109	-0.12	-0.05	0.131	.372**	-0.114	-.212**	.174*	.260**	-0.105	.466**	1							
⑱ Cre	.268**	.420**	.382**	.442**	.442**	-.504**	-.529**	-.281**	.170*	.351**	-.227**	-.483**	.494**	.452**	0.001	.352**	.376**	1						
⑲ Dialysis time	.193*	.238**	.222**	.216**	.240**	-0.134	-0.126	-0.106	0.045	.206**	.451**	-.248**	.349**	0.082	-0.095	.266**	0.005	0.108	1					
⑳ BUN	0.093	0.137	0.124	.196*	.163*	-.190*	-.167*	-.178*	0.115	.191*	0.083	-0.063	.333**	0.116	0.072	.183*	0.13	.299**	.154*	1				
㉑ HDL-c	-0.013	0.056	0.014	0.028	0.03	.155*	.174*	0.062	0.002	-0.04	.460**	0.021	-.247**	-.437**	0.088	0.114	-0.059	-.212**	.181*	0.064	1			
㉒ LDL-c	-0.02	0.039	0.017	0.026	0.027	-0.007	-0.031	0.048	-0.015	-0.07	.260**	-.183*	0.029	0.047	0.122	0.039	.236**	.158*	0.105	.222**	0.055	1		
㉓ Right ba PWV(cm/s)	-0.061	-.304**	-.225**	-.212**	-.256**	.191*	.221**	0.061	-0.083	-0.084	-0.072	.373**	-.164*	-.178*	-0.046	-.161*	-0.078	-.333**	-0.084	-0.146	-0.046	-.199*	1	
㉔ Left ba PWV(cm/s)	-0.098	-.388**	-.253**	-.259**	-.308**	0.134	.163*	0.027	-0.045	-0.064	-0.104	.382**	-0.153	-0.146	0.008	-.164*	-0.073	-.265**	-0.108	-0.111	-0.073	-.207**	.877**	1

LSA, Life Space Assessment; KCL, Kihon Check List; ABI, ankle-brachial pressure index; GNRI, Geriatric Nutritional Risk Index; Kt/V, K reflects clearance of BUN, t reflects dialysis time, and V reflects body fluid volume; BMI, body mass index; Alb, albumin; Hb, hemoglobin; Cre, creatinine; BUN, blood urea nitrogen; HDL-c, high density lipoprotein cholesterol; LDL-c, low density lipoprotein cholesterol; ba PWV, brachial-ankle pulse wave velocity. *p<0.05 , ** p<0.01
